# Supplementary material for: Comparative demography of commercially-harvested snappers and an emperor from American Samoa
Source: PeerJ. 2018 Jun 18;6:e5069. doi: 10.7717/peerj.5069 (PMC6011874; doi:10.7717/peerj.5069)
Supplement: Supplemental Information 1 [file peerj-06-5069-s001.docx]

**Supplemental Information**

Comparative demography of commercially-harvested snappers and an emperorfish from American Samoa

Brett M. Taylor, Zack S. Oyafuso, Cassandra Pardee, Domingo Ochavillo, Stephen J Newman

Table S1. Sensitivity analysis of length bin width (for all years combined) and year (using 5-cm size bins) on the median and 95% confidence interval of total mortality rate for harvested populations of *Lethrinus xanthochilus*, *Lutjanus gibbus*, and *Lutjanus rufolineatus* from Tutuila, American Samoa. Too few samples were surveyed for *L. rufolineatus* to run the analyses.

|  | *Lethrinus xanthochilus* | | |  | *Lutjanus gibbus* | | |  | *Lutjanus rufolineatus* | | |
| --- | --- | --- | --- | --- | --- | --- | --- | --- | --- | --- | --- |
| Bin Size | 2.5% | 50% | 97.5% |  | 2.5% | 50% | 97.5% |  | 2.5% | 50% | 97.5% |
| 2 cm | 0.30 | **0.36** | 0.41 |  | 0.21 | **0.24** | 0.28 |  | 0.44 | **0.53** | 0.70 |
| 5 cm | 0.29 | **0.35** | 0.41 |  | 0.19 | **0.22** | 0.26 |  | 0.44 | **0.54** | 0.75 |
| 10 cm | 0.30 | **0.36** | 0.43 |  | 0.19 | **0.22** | 0.26 |  | 0.44 | **0.53** | 0.74 |
| 20 cm | 0.30 | **0.36** | 0.44 |  | 0.20 | **0.24** | 0.28 |  | 0.45 | **0.54** | 0.75 |
| 2011 | 0.32 | **0.39** | 0.46 |  | 0.21 | **0.24** | 0.28 |  | 0.43 | **0.52** | 0.74 |
| 2012 | 0.32 | **0.39** | 0.47 |  | 0.21 | **0.24** | 0.28 |  | 0.43 | **0.52** | 0.74 |
| 2013 | 0.32 | **0.39** | 0.46 |  | 0.21 | **0.24** | 0.28 |  | 0.43 | **0.52** | 0.74 |
| 2014 | 0.32 | **0.39** | 0.46 |  | 0.21 | **0.24** | 0.28 |  | NA | **NA** | NA |
| 2015 | 0.33 | **0.39** | 0.46 |  | 0.21 | **0.24** | 0.28 |  | 0.43 | **0.52** | 0.74 |

Table S2. Proportions of opaque margins on the outer edge of transverse otolith sections by month by species. Sample sizes by month and species are denoted in superscripts.

|  | Proportion of opaque margins*^sample size^* | | |
| --- | --- | --- | --- |
|  | *L. xanthochilus* | *L. gibbus* | *L. rufolineatus* |
| Jan | 0*^16^* | 0*^14^* | 0.0667*^15^* |
| Feb | 0*^13^* | 0*^14^* | 0*^16^* |
| Mar | 0.1667*^24^* | 0.1818*^11^* | 0.1053*^19^* |
| Apr | 0.0714*^14^* | 0.1176*^17^* | - |
| May | 0.1875*^16^* | 0.2*^5^* | - |
| Jun | - | - | - |
| Jul | 0.2*^5^* | 0.3333*^6^* | 0.4286*^7^* |
| Aug | 0.4286*^7^* | 0.4*^10^* | 0.5*^12^* |
| Sep | - | 0.4*^5^* | 0.25*^4^* |
| Oct | 0.2105*^19^* | 0.3514*^37^* | 0.25*^24^* |
| Nov | 0*^11^* | 0.0588*^17^* | 0*^12^* |
| Dec | 0.0476*^21^* | 0*^18^* | 0.1667*^6^* |


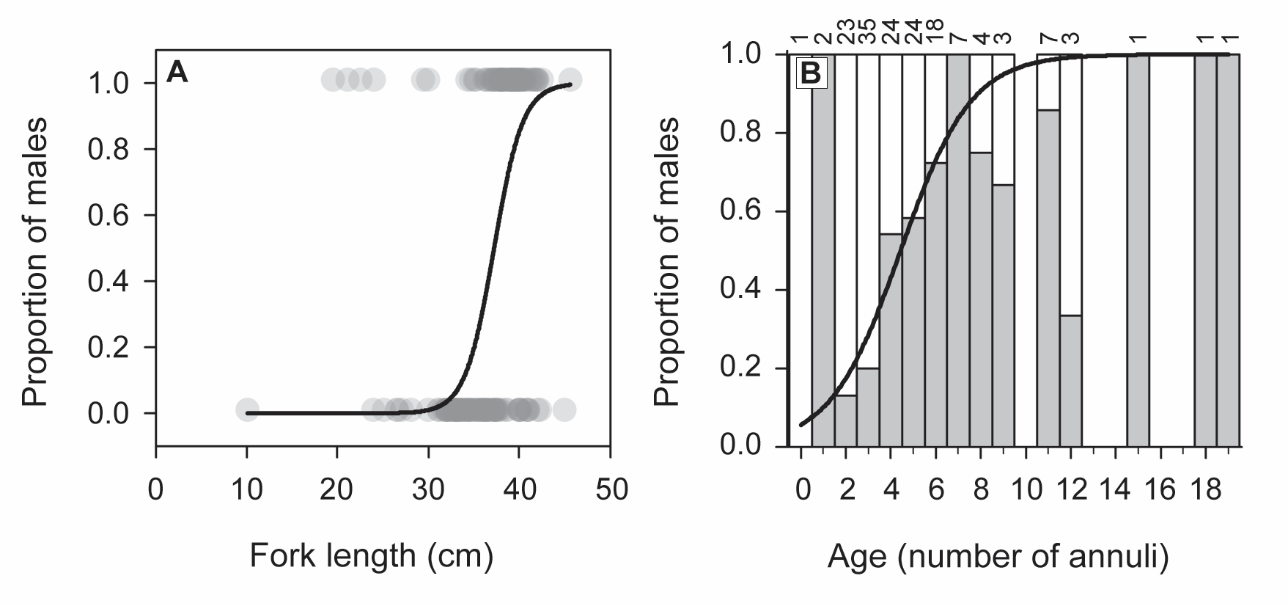


Figure S1. Modelled schedules of male:female sex ratio by (A) fork length and (B) age class, as evidence for potential postmaturational female-to-male sex change. Curves were fitted using logistic models following the length at maturity estimation in the main text. Numbers above age class bins in (B) represent sample sizes for each age class.
